# Trends in survival of patients with stage I/II Burkitt lymphoma in the United States: A SEER database analysis

**DOI:** 10.1002/cam4.1870

**Published:** 2019-01-31

**Authors:** Ze‐Long Liu, Pan‐Pan Liu, Xi‐Wen Bi, De‐Xin Lei, Yu Wang, Zhi‐Ming Li, Wen‐Qi Jiang, Yi Xia

**Affiliations:** ^1^ Department of Medical Oncology, State Key Laboratory of Oncology in South China, Collaborative Innovation Centre of Cancer Medicine Sun Yat‐sen University Cancer Center Guangzhou People’s Republic of China

**Keywords:** Burkitt lymphoma, rituximab, SEER analysis, survival

## Abstract

The treatment strategy for management of Burkitt lymphoma (BL) has evolved during the past decades and the clinical outcome for this disease as a whole has also improved. Due to limited information reported on survival trends of patients with stage I/II (limited‐stage) BL, here we used the Surveillance, Epidemiology, and End Results (SEER) database to conduct our study. The time period was divided into two eras (1983‐2001 and 2002‐2014) as the recent era reflected more intensive chemotherapy regimens, the availability of rituximab, the widespread use of antiretroviral therapy (ART) and improvements in supportive care. Patients with limited‐stage BL had a significantly better 5‐year overall survival (OS) in the 2002‐2014 era in both univariate analysis and multivariate analysis, compared with those in the 1983‐2001 era (64.1% vs 57.4%). However, clinical outcomes of elderly patients (≥60 years) and children patients (0‐19 years) did not significantly improve. Older age and race of black were correlated with poorer OS in multivariate analysis, whereas sex, primary sites, and application of radiotherapy did not significantly influence OS. In conclusion, the prognosis of patients with limited‐stage BL has improved in the 2002‐2014 era, but the outcome was still much poorer in elderly patients, which needs to be improved by identifying newly molecular‐targeted drugs and developing novel personalized therapeutic approaches.

## 1 INTRODUCTION

Burkitt lymphoma (BL) is a highly aggressive B‐cell non‐Hodgkin lymphoma (NHL) characterized by the translocation and deregulation of the MYC gene on chromosome 8. There are three distinct clinical types of BL: endemic BL, sporadic BL, and immunodeficiency‐related BL.^1^, ^2^, ^3^, ^4^ Although differing in epidemiology, clinical manifestations, and genetic features, they are histologically identical and have similar clinical behavior. All three clinical variants are generally treated in a similar fashion.

Less intensive regimens like CHOP are inadequate therapy for BL as they result in high recurrence rates.^5^, ^6^ Intensive, multi‐agent combination chemotherapy with adequate central nervous system (CNS) prophylaxis yields excellent outcomes.^7^, ^8^, ^9^, ^10^, ^11^ The addition of rituximab, an anti‐CD20 monoclonal antibody, to the combination chemotherapy has been confirmed to improve outcomes of some subtypes of B‐cell NHLs. Several uncontrolled, prospective trials and one randomized trial also suggested that the incorporation of rituximab in the management of BL could improve event‐free survival (EFS) and/or overall survival (OS), with 2‐year OS rates from 77% to 100%.^12^, ^13^, ^14^, ^15^, ^16^, ^17^, ^18^, ^19^, ^20^

Clinically, patients with BL generally present with an advanced stage (stage III/IV) at diagnosis and with rapidly growing tumor masses. There is a small subset of BL presents in stage I and II (limited stage) and this small population has a better outcome than those with advanced stage.^20^ The outcome of patients with advanced stage has improved^21^ in the recent era owing to the use of more intense chemotherapy regimens,^7^, ^8^, ^9^, ^10^ the availability of rituximab,^7^, ^12^, ^14^, ^16^, ^17^, ^22^, ^23^, ^24^, ^25^, ^26^, ^27^, ^28^, ^29^, ^30^ the application of antiretroviral therapy (ART),^31^ and improvements in supportive care.^9^, ^10^ However, it is unclear whether the recent changes in management modalities have affected the survival of limited‐stage BL patients who already had excellent cure rates. In this study, we aimed to provide a better understanding of trends in survival of patients with stage I/II BL in the United States, through using the Surveillance, Epidemiology, and End Results (SEER) database. We also aimed to determine the survival trends of different patient groups and identify factors that influenced the prognosis.

## 2 MATERIALS AND METHODS

### 2.1 Inclusion of patients

The SEER database collects and publishes data of cancer incidence, treatment, and survival from population‐based cancer registries, covering about 28% of the USA population. We extracted data of patients with stage I/II BL from the SEER‐18 to conduct this analysis. The SEER‐18 registry includes Atlanta, Detroit, Greater California, Greater Georgia, Hawaii, Iowa, Kentucky, Los Angeles, New Mexico, New Jersey, Rural Georgia, states of Connecticut, San Francisco‐Oakland, Seattle‐Puget Sound, San Jose Monterey, the Alaska Native Tumor Registry, Louisiana, and Utah.

**Figure 1:**
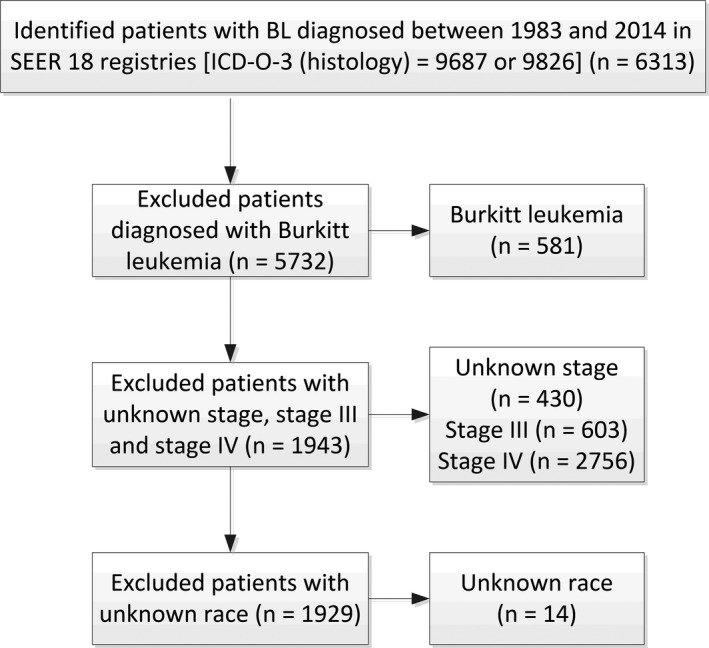
Flowchart of patient inclusion

The third edition of the International Classification of Disease for Oncology (ICDO‐3) is used to identify BL in the SEER database (codes 9687 and 9826). The inclusion period in this study is from 1983 to 2014 as the Ann Arbor staging system was widely used for lymphomas since 1983. Exclusion criteria included patients with Burkitt leukemia, or with unknown stage, stage III, stage IV, or with unknown race, or received no chemotherapy (Figure 1). Characteristics of patients, including age, sex, race, stage, primary sites, and with/without radiotherapy (RT) were extracted. Patients were divided into two eras (the 1983‐2001 era and the 2002‐2014 era) based on the year of diagnosis. The period of 2002‐2014 was expected to reflect the effects of more intense chemotherapy regimens, the availability of rituximab, the widespread use of ART, and potential improvements in supportive care on the improvement of prognosis of BL patients.

### 2.2 Statistical analysis

Statistical analysis was conducted through using the Statistical Package for the Social Sciences (SPSS) version 20.0 software (IBM Corporation, Armonk, NY) Categorical variables including age, sex, race, stage, primary sites, and with/without radiotherapy were compared by chi‐squared tests. Survival analysis was performed by plotting Kaplan‐Meier survival curves, in which log‐rank tests were utilized to detect statistically significant differences. Overall survival (OS) was defined as the time from diagnosis to death from any cause. The impact of the year of diagnosis, age, sex, race, stage, primary sites, and radiotherapy on the OS was studied through univariate and multivariate analysis using the Cox proportional hazard regression method. A *P *value of <0.05 was considered statistically significant for all statistical analyses.

## 3 RESULTS

A total of 1929 patients with stage I/II BL from the SEER database were included in this study, 539 (27.9%) in the 1983‐2001 era and 1390 (72.1%) in the 2002‐2014 era. The median age at diagnosis was 40 years (range: 0‐101 years) and the male to female ratio was 2.98:1. 82.5% of included patients were whites; over half of them were classified as stage I (56.6%) and had primary tumor in lymph nodes (53.6%). Only 11.8% of patients received radiotherapy. Comparisons of characteristics of patients in two eras were presented in Table 1. There were no statistically significant differences between the two eras in terms of sex and race. Conversely, significant differences between the two eras were found in terms of age group, stage, primary sites, and application of radiotherapy.

**Table 1:** Demographic and clinical characteristics of patients with stage I/II BL in the 1983‐2001 era and the 2002‐2014 era

Variable	Total (N = 1929)	Era		*P* value^a^
		1983‐2001 (n = 539; 27.9%)	2002‐2014 (n = 1390; 72.1%)	
Age group				
0‐19 y	518 (26.9%)	171 (31.7%)	347 (25.0%)	**0.004**
20‐39 y	427 (22.1%)	127 (23.6%)	300 (21.6%)	
40‐59 y	506 (26.2%)	121 (22.4%)	385 (27.7%)	
≥60 y	478 (24.8%)	120 (22.3%)	358 (25.8%)	
Sex				
Male	1440 (74.7%)	407 (75.5%)	1033 (74.3%)	0.600
Female	489 (25.3%)	132 (24.5%)	357 (25.7%)	
Race^b^				
White	1592 (82.5%)	456 (84.6%)	1136 (81.7%)	0.289
Black	158 (8.2%)	41 (7.6%)	117 (8.4%)	
Others	179 (9.3%)	42 (7.8%)	137 (9.9%)	
Stage^a^				
I	1092 (56.6%)	329 (61.0%)	763 (54.9%)	**0.016**
II	837 (43.4%)	210 (39.0%)	627 (45.1%)	
Primary sites				
Nodal	1034 (53.6%)	314 (58.3%)	720 (51.8%)	**0.011**
Extranodal	895 (46.4%)	225 (41.7%)	670 (48.2%)	
Radiotherapy				
Yes	228 (11.8%)	111 (20.6%)	117 (8.4%)	**<0.001**
No	1701 (88.2%)	428 (79.4%)	1273 (91.6%)	

a Statistically significant results are shown in bold.

b Analysis excludes unknown or missing values.

**Figure 2:**
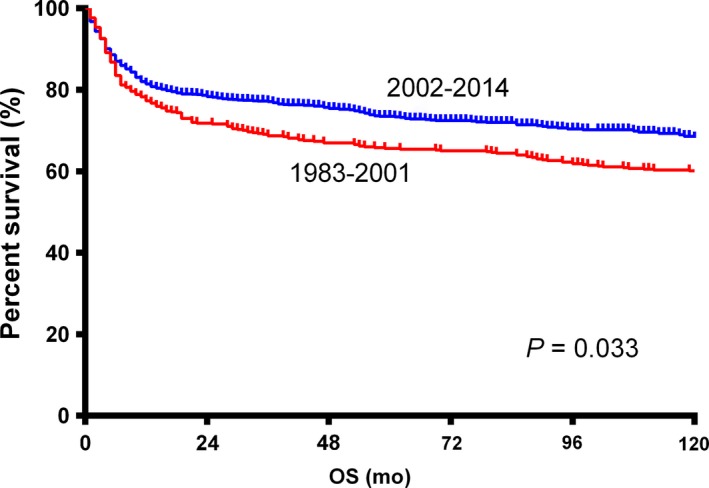
Kaplan‐Meier survival curves of BL patients in the 1983‐2001 era and the 2002‐2014 era

The 5‐year OS for the whole population was 62.0%. Patients in the 2002‐2014 era had a significantly better OS than those in the 1983‐2001 era (HR = 0.839, 95% CI: 0.714‐0.986, *P = *0.033; Figure 2). Survival curves through the Kaplan‐Meier analysis indicated no significant differences between groups of different races, stage I and II, and primary nodal and extranodal BL (Table 2). Elderly patients, female patients, and patients treated with RT had significantly poorer OS (Table 2).

**Table 2:** Univariate and multivariable analyses summarizing associations with BL overall survival

Variable	5‐y OS (%)	Univariate analysis		Multivariate analysis	
		HR (95% CI)	*P* a	HR (95% CI)	*P* a
Era					
1983‐2001	57.4%	1		1	
2002‐2014	64.1%	0.839 (0.714‐0.986)	**0.033**	0.746 (0.634‐0.877)	**<0.001**
Age					
0‐19 y	92.6%	1		1	
20‐39 y	69.6%	4.734 (3.279‐6.835)	**<0.001**	4.819 (3.337‐6.959)	**<0.001**
40‐59 y	56.2%	7.834 (5.531‐11.096)	**<0.001**	8.229 (5.806‐11.664)	**<0.001**
≥60 y	26.7%	16.811 (11.949‐23.653)	**<0.001**	17.766 (12.617‐25.017)	**<0.001**
Sex					
Female	56.3%	1		1	
Male	64.0%	0.753 (0.640‐0.886)	**0.001**	1.043 (0.883‐1.232)	0.622
Race					
Black	57.8%	1		1	
White	62.7%	0.842 (0.649‐1.093)	0.196	0.684 (0.526‐0.889)	**0.005**
Others	59.4%	0.934 (0.661‐1.319)	0.698	0.636 (0.450‐0.901)	**0.011**
Stage					
I	62.7%	1		1	
II	61.2%	1.096 (0.943‐1.274)	0.234	1.173 (1.008‐1.365)	**0.039**
Primary site					
Nodal	63.5%	1		1	
Extranodal	60.1%	1.084 (0.933‐1.259)	0.291	1.158 (0.994‐1.349)	0.060
Radiotherapy					
No RT	64.4%	1		1	
RT	46.3%	1.620 (1.333‐1.968)	**<0.001**	1.124 (0.920‐1.372)	0.252

a Statistically significant results are shown in bold.

Using multivariate analysis (Table 2), we found that the 2002‐2014 era was independently associated with a significantly better OS, compared with the 1983‐2001 era (*P < *0.001). Age ≥60 years was associated with a significantly poorer OS in multivariate analysis, which was consistent with the conclusion of univariate analysis. Race of black and stage II were both independently correlated with poorer OS. No statistically significant difference was detected between the OS of patients with primary nodal and extranodal BL (*P = *0.060). Differing from results of the univariate analysis, there was no significant difference between the OS of male and female (*P = *0.622), or RT and non‐RT (*P = *0.252).

**Figure 3:**
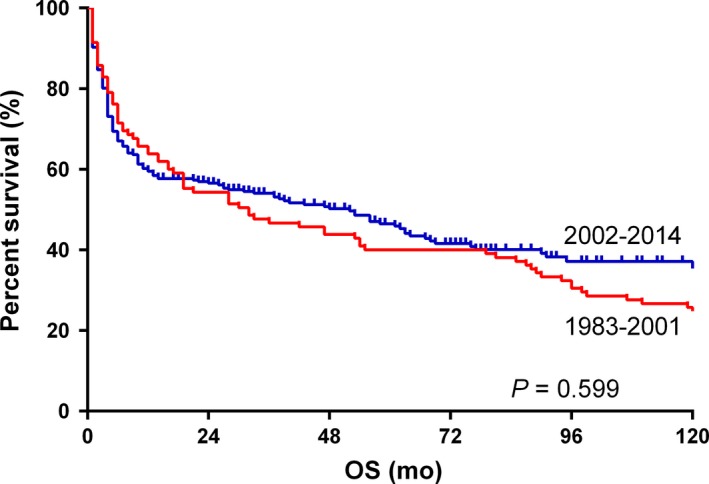
Kaplan‐Meier survival curves of elderly BL patients (≥60) in the 1983‐2001 era and the 2002‐2014 era

In subgroup analyses, we found that there was no significant difference between the OS of elderly patients (≥60 years) in two eras (*P = *0.599; Figure 3). A similar result was observed in the 0‐19 years group, whereas patients of the other two groups (the 20‐39 years group and the 40‐59 years group) in the 2002‐2014 era had significantly better OS than those in the 1983‐2001 era. White patients had a better OS in the 2002‐2014 era (Figure S1), whereas black patients and patients of other races did not show a similar result. A better OS was found in male patients in the 2002‐2014 era (Figure S2). Results of subgroup analyses were summarized in Table 3.

**Table 3:** Comparing OS of BL patients between the 1983‐2001 era and the 2002‐2014 era in different groups

Variable	1983‐2001 era		2002‐2014 era		*P* value^a^
	N	HR (95% CI)	N	HR (95% CI)	
Age					
0‐19 y	171	1	347	0.880 (0.448‐1.727)	0.710
20‐39 y	127	1	300	0.450 (0.311‐0.651)	**<0.001**
40‐59 y	121	1	385	0.688 (0.512‐0.924)	**0.013**
≥60 y	120	1	358	0.937 (0.734‐1.196)	0.599
Sex					
Female	132	1	357	0.883 (0.654‐1.193)	0.417
Male	407	1	1033	0.815 (0.673‐0.987)	**0.036**
Race					
Black	41	1	117	0.877 (0.509‐1.512)	0.637
White	456	1	1136	0.835 (0.698‐0.999)	**0.048**
Others	42	1	137	0.807 (0.477‐1.367)	0.425
Primary site					
Nodal	314	1	720	0.822 (0.659‐1.026)	0.082
Extranodal	225	1	670	0.847 (0.668‐1.074)	0.170

a Statistically significant results are shown in bold.

## 4 DISCUSSION

Limited stage (stage I and II) is a rare presentation of BL. According to previous studies based on the SEER database, about 36%‐38.4% of patients with BL were reported to have a limited stage at diagnosis.^21^, ^32^ The majority of patients with BL may be cured with aggressive treatment regimens, while patients with limited stage have an even better outcome. Whether the excellent survival in this small cohort is further improved with the advancement in dose intensification schedules, the introduction of the rituximab, the application of antiretroviral therapy, and improvements in supportive care is unknown currently. Our present study, for the first time, reveals that significant survival improvements could be observed in patients with limited stage diagnosed in the recent era.

Three main treatment approaches have been used for the management of BL, including intensive, short‐duration combination chemotherapy like R‐CODOX‐M/IVAC (“Magrath regimen”), infusional chemotherapy with dose‐adjusted EPOCH (DA‐EPOCH) plus rituximab, and ALL‐like therapy with a stepwise induction, consolidation, and maintenance therapy lasting at least 2 years from diagnosis represented by Hyper‐CVAD/MA regimen. The initial study of CODOX‐M/IVAC from the National Cancer Institute (NCI) in 41 newly diagnosed BL reported a 2‐year EFS of 92%.^9^ A prospective multicenter study focused on the use of a risk‐adapted CODOX‐M/IVAC protocol in 52 de novo BL patients observed that the 2‐year OS rates for the low‐ and high‐risk patients were 82% and 70%, respectively.^33^ Another prospective, nonrandomized trial consisting of 53 patients with newly diagnosed BL treated with a risk‐adjusted, dose‐modified CODOX‐M/IVAC protocol reported the 2‐year progression‐free and overall survival rates of 64% and 67%, respectively.^7^ The DA‐EPOCH regimen has been evaluated in single‐arm prospective trials. In the prospective study consisting of 17 BL patients receiving R‐DA‐EPOCH therapy, the estimated rate of OS at 7 years was 100% after a median follow‐up of 86 months.^26^ A retrospective study included 26 adults with newly diagnosed BL treated with Hyper‐CVAD regimen showed the 3‐year OS rate was 49%.^8^ And another prospective, single‐arm study evaluated Hyper‐CVAD plus rituximab alternating with high‐dose methotrexate and cytarabine in 31 newly diagnosed BL patients showed a 3‐year OS rate of 89%.^12^

The addition of rituximab into chemotherapy regimens is another practical way to improve the therapeutic effect. Rituximab is an anti‐CD20 monoclonal antibody that has been proved effective in most B‐cell NHLs and also showed promising results in BL. The strongest evidence that supported incorporation of rituximab in the management of BL is from a multicenter, randomized, controlled phase III clinical study,^20^ in which the addition of rituximab significantly improved the 3‐year EFS from 62% to 75% and the 3‐year OS from 70% to 83%. Nonrandomized trials further supported that the addition of rituximab resulted in high survival rates and low toxicity. The largest prospective, multicenter single‐arm trial consisting of 363 BL patients received short‐duration intensive combination chemotherapy with rituximab reported that the estimated OS at 5 years was 80%.^13^

Another concern during treatment is the HIV infection status since approximately 20% of BL patients were HIV‐infected patients in the current United States. As reported, HIV‐infected BL patients treated with current chemotherapy regimens had a 2‐year EFS of over 60%.^33^ The widespread use of ART since 2001 has decreased the incidence of NHLs but not BL in HIV‐infected patients.^34^ A PETHEMA study of 18 patients found that ART prolonged the OS of HIV‐infected BL patients treated with chemotherapy regimens.^31^

In this study, we selected total 1929 patients with stage I/II BL diagnosed between 1983 and 2014, since the time when the Ann Arbor staging system for BL was widely used. Included patients in our study were divided into two eras, the 1983‐2001 era and the 2002‐2014 era. Our study found the improved long‐time survival of BL patients with a limited stage in the 2002‐2014 era, with a 5‐year OS rate of 64.1%.

In this study, the 5‐year OS rate was over 90% for children patients (0‐19 years) with limited‐stage BL, which is consistent with results of previously published literature (Table 2). Five‐year OS rates declined with increasing age being 69.6%, 56.2%, and 26.7% for patients of 20‐39 years, 40‐59 years, and ≧60 years, respectively (Table 2). However, HIV infection prevalence is not independent from age. According to Shiels MS's study, proportions of BL cases with acquired immune deficiency syndrome (AIDS) in 0‐29 years, 30‐59 years, and ≥60 years age groups were 7.8%, 40.3%, and 1.7%, respectively.^34^ Thus, the HIV infection and its treatment mainly influenced prognosis of BL in the 20‐39 and 40‐59 age groups in our study. Although the age of patients was older in the 2002‐2014 era (*P = *0.004), a significantly better 5‐year OS of patients in this era than those in the 1983‐2001 era was observed in both univariate analysis and multivariate analysis, with *P* values of 0.033 and <0.001, respectively (Table 2). However, subsequent subgroup analyses of two eras found that both 0‐19 years and ≥60 years patients did not show a better OS in the 2002‐2014 era, while both 20‐39 years and 40‐59 years patients did (Table 3). As mentioned above, due to a much larger proportion of HIV‐infected patients, HIV infection treatment also contributed to prolonged OS of the 20‐39 years age group and the 40‐59 years age group. However, lack of information on HIV infection status would not enable us to further analyze whether the improved chemotherapy regimens or the improved HIV infection treatment plays a more important role.

Patients of black race exhibited a significantly poorer prognosis than patients of white race and other races. Besides, unlike patients of white race, patients of black race and other races did not exhibit better clinical outcomes in the 2002‐2014 era. Stage II was significantly associated with poorer OS in multivariate analysis (HR = 1.173, 95% CI: 1.008‐1.365, *P = *0.039), compared with stage I (Table 2). As shown in Table 1, we found that the 2002‐2014 era contained more stage II patients (*P = *0.016). Despite this, patients in the 2002‐2014 era still had better clinical outcomes, which further confirmed our previous conclusion. On the contrary, sex and primary sites of the tumor (nodal or extranodal) did not significantly influence the OS of these patients (Table 2). However, male patients but not female patients were observed to have a significantly better prognosis in the 2002‐2014 era.

We also found that the application of RT in patients with limited‐stage BL did not improve the OS. Conversely, patients treated with RT seemed to have poorer OS, although the difference was not statistically significant (HR = 1.124, 95% CI: 0.920‐1.372, *P = *0.252). An explanation of this interesting result is that RT is only applied in a small number of patients with bulky disease, as bulky disease is known to have a worse prognosis. The proportion of patients with limited‐stage BL that received RT significantly decreased in the 2002‐2014 era, compared with those in the 1983‐2001 era (8.4% vs 20.6%, *P* < 0.001). These results suggested that patients with limited‐stage BL did not benefit from RT and should not be regularly treated with RT.

This study had some unavoidable limitations that should be considered when interpreting our results. First of all, chemotherapy regimens of included patients were unclear, which led the difficulty in determining the percentage of patients that received rituximab therapy and assessing the comparability of chemotherapy regimens between two eras. Secondly, some B‐cell lymphomas classified as BL in the past are now classified as diffuse large B‐cell lymphoma or “B‐cell lymphoma, unclassifiable, with features intermediate between diffuse large B‐cell lymphoma and Burkitt lymphoma.” These lymphomas might have translocations involving c‐myc, bcl2, or bcl6 (named double‐hit or triple‐hit lymphomas) and have a worse prognosis than BL. Lack of centralized pathology review and centralized imaging review also caused unavoidable errors of pathological diagnosis and staging of some patients. Additionally, important detailed information of the HIV status was not available in the SEER database. HIV incidence in the United States increased in the mid‐1990s, then slightly declined after 1999 and has been stable thereafter, according to published statistics from The Centers for Disease Control and Prevention.^35^, ^36^, ^37^ However, according to a published paper, proportions of BL cases with HIV infection in 1980‐1989, 1990‐1995, 1996‐2000, and 2001‐2007 were 13.1%, 27.4%, 16.0%, and 20.8%, respectively.^34^ Thus, it seems that the latter period of our study had a similar proportion of HIV + BL patients compared to that in the earlier period. As there is no HIV infection information in the SEER database, we could not analyze the impact of HIV infection on outcome in any period. HIV‐infected individuals had a different OS in the two eras. HIV‐infected BL represented about 20% of whole BL population, the specific impact of this small population on the outcome of BL in the two periods were unknown. Also, we could not analyze the association of HIV infection and age segments. Also, patients with incomplete important information were roughly excluded in this study. The registries were more complete in the second period, which could partially explain the obvious difference between patient numbers in the two eras. Rapid developments of pathological and molecular techniques of diagnosis for BL are also somehow related to the increased incidence of BL.

As far as we know, this is the first study to comprehensively analyze trends in survival for patients with stage I/II BL. In both univariate and multivariate analyses, we found a significant improvement of the OS in the 2002‐2014 era. However, the prognosis of elderly patients (≥60 years) remained much worse than children patients in the rituximab era and further efforts still need to be made. Considering the limitations of this study, well‐designed clinical randomized studies should be performed to confirm our results in the future.

## CONFLICT OF INTEREST

No conflict of interest to be declared.
